# Bayesian inference for biomarker discovery in proteomics: an analytic solution

**DOI:** 10.1186/s13637-017-0062-4

**Published:** 2017-07-14

**Authors:** Noura Dridi, Audrey Giremus, Jean-Francois Giovannelli, Caroline Truntzer, Melita Hadzagic, Jean-Philippe Charrier, Laurent Gerfault, Patrick Ducoroy, Bruno Lacroix, Pierre Grangeat, Pascal Roy

**Affiliations:** 1 IMS (Univ. Bordeaux, CNRS, BINP), Talence, 33400 France; 2 National Engineering School of Gabes (ENIG), University of Gabes, Gabes, Tunisia; 3 CLIPP, Pôle de Recherche Université de Bourgogne, Dijon, 21000 France; 4 NATO STO Centre for Maritime Research and Experimentation, La Spezia, 19126 Italy; 5 Technology Research Department, Innovation Unit, bioMérieux SA, Marcy l’Étoile, France; 6 Univ. Grenoble Alpes, Grenoble, F-38000 France; 7 CEA, LETI, MINATEC Campus, Grenoble, F-38054 France; 8 Service de Biostatistique - Bioinformatique, Hospices Civils de Lyon, Lyon, France; 9 CNRS UMR 5558, LBBE, Équipe Biostatistique Santé, Villeurbanne, France; 10 Université de Lyon, Université Claude Bernard Lyon 1, Lyon, France; 11 Pôle Rhône-Alpes de Bioinformatique, Université Claude Bernard - Lyon 1, Villeurbanne, 69622 France

**Keywords:** Variable selection, Model selection, Optimal decision, Bayesian approach, Evidence, Hierarchical model, Proteomics, Biomarker

## Abstract

This paper addresses the question of biomarker discovery in proteomics. Given clinical data regarding a list of proteins for a set of individuals, the tackled problem is to extract a short subset of proteins the concentrations of which are an indicator of the biological status (healthy or pathological). In this paper, it is formulated as a specific instance of variable selection. The originality is that the proteins are not investigated one after the other but the best partition between discriminant and non-discriminant proteins is directly sought. In this way, correlations between the proteins are intrinsically taken into account in the decision. The developed strategy is derived in a Bayesian setting, and the decision is optimal in the sense that it minimizes a global mean error. It is finally based on the posterior probabilities of the partitions. The main difficulty is to calculate these probabilities since they are based on the so-called evidence that require marginalization of all the unknown model parameters. Two models are presented that relate the status to the protein concentrations, depending whether the latter are biomarkers or not. The first model accounts for biological variabilities by assuming that the concentrations are Gaussian distributed with a mean and a covariance matrix that depend on the status only for the biomarkers. The second one is an extension that also takes into account the technical variabilities that may significantly impact the observed concentrations. The main contributions of the paper are: (1) a new Bayesian formulation of the biomarker selection problem, (2) the closed-form expression of the posterior probabilities in the noiseless case, and (3) a suitable approximated solution in the noisy case. The methods are numerically assessed and compared to the state-of-the-art methods (*t* test, LASSO, Battacharyya distance, FOHSIC) on synthetic and real data from proteins quantified in human serum by mass spectrometry in selected reaction monitoring mode.

## Introduction

It is now generally recognized that protein expression analysis is crucial in explaining the changes that occur as a part of disease pathogenesis [1, 2]. In this context, recent advances in mass spectrometry (MS) technologies have facilitated the investigation of proteins over a wide range of molecular weights in small biological specimens from blood or urine samples, for instance. Notably, MS in selected reaction monitoring (SRM) mode has demonstrated its ability to quantify clinical biomarkers in patient sera [3, 4]. Consequently, a large amount of research has been generated in proteomics based on data such as protein mass spectral intensities or protein concentrations obtained from the spectra. Specifically, the focus is on the selection (or discovery) of the “signature profiles,” the so-called biomarkers. They represent, for instance, indicators of normal versus pathogenic biological processes, or positive versus negative pharmacological responses to therapeutic intervention.

Critical to the identification of biomarkers are: (1) the biological variability, i.e., the random variations of the concentrations of proteins between individuals sharing the same biological status [5], and (2) the technical variability, which originates from the imperfections of the measurement process used to obtain the concentrations. Failing to address both of these variabilities within a technique for biomarker identification may significantly impair its performance by resulting in erroneous decision.

Furthermore, since the complexity of a status is unlikely to be manifested through the changes in the characteristics of just one protein, it has generally been acknowledged that a set of proteins should be considered [5–8]. An additional difficulty is that they are possibly correlated, imposing the use of multivariate models to account for all the data simultaneously. These aforementioned issues pose significant challenges in developing efficient and robust statistical techniques for the identification of biomarkers.

The paper tackles the problem of biomarker identification by adopting a Bayesian approach to propose the selection of the optimal set of variables. By providing an elegant and mathematically rigorous framework for incorporating the data and the prior information within a joint probabilistic model, the Bayesian setting allows straightforward modeling of both the technical and the biological variabilities of the data.

The remainder of the paper is organized as follows. Section 2 summarizes the state-of-the-art variable selection methods, discusses their main challenges, and outlines our principal contributions. Section 3 presents the proposed formulation within the Bayesian framework, the proposed models for the data, and the decision strategy. Section 4 describes the data used in the numerical evaluations, together with the results and their analysis. Finally, conclusions are drawn in Section 5. A detailed description of the model and the derivation of the analytic solution is provided in Appendix.

## Related work

The identification of biomarkers for diagnosis or prognosis can be classically formulated as a variable selection problem, and this problem has been paid a lot of attention as a specific instance of model choice. Various methodologies exist that can be broadly classified in two categories: the frequentist hypothesis testing and the Bayesian decision-making.

Frequentist hypothesis testing consists in deciding between two statements, classically referred to as the null and the alternative hypotheses, by comparing a function of the observed data to a threshold. The reader is invited to consult [9] for a comprehensive overview. Two closely related methods have been proposed. On the one hand, Neyman-Pearson tests are designed to ensure the so-called type I error. On the other hand, *p* values focus on how strongly the data reject the null hypothesis *H*_0_ by evaluating the probability of obtaining a value as extreme as the observed one given *H*_0_ is true. In biomarker discovery, a popular approach consists in testing a mean difference between the case and the negative controlled populations using the classical Students’ *t* test or its variants [7]. The latter is a statistical hypothesis test which indicates whether the difference between two group means most likely reflects that they are samples of two different populations or, on the contrary, is merely explained by the sampling fluctuation. However, as the number of candidate biomarkers increases, multiple hypothesis testing is required resulting in a higher computational cost which may become prohibitive [10]. A first solution is to perform univariate tests for each protein.

This procedure requires an adapted control of the rate of type I errors in this particular setting where multiple hypothesis tests are conducted simultaneously. Two types of procedures were proposed for this purpose, namely the so-called family wise error rate or the false discovery rate [11, 12]. A common criticism of frequentist approaches is that they fail to take into account prior information about the problem at hand such as interdependencies between the different variables.

For a large number of candidate variables, regression analysis [13] provides an alternative to the above-mentioned methods. The principle is to assume that a given outcome is related to a linear combination of a set of explanatory variables called the predictors. In proteomics, logistic regression models are considered that express the probability to have a disease as a function of the protein abundances [14–16]. Then, variable selection is classically performed using stepwise procedures that consists in successively adding or removing predictors, estimating the regression coefficients, and evaluating the goodness of fit of the subsequent model. Different criteria can be considered such as the *R*-squared, the adjusted *R*-squared, or the Akaike Information Criterion [17, 18]. Such techniques are referred to as backward elimination and forward selection, respectively [13]. However, these selection procedures are prone to overfitting and the variance of the parameter estimates becomes high in the presence of correlated predictors. Regularization methods alleviate these difficulties by considering the minimum of a penalized least squares error as estimate. Since the Ridge regression in 1970 [19], several algorithms have been proposed that differ between one another with respect to the considered penalization of the regression parameters. The well-known LASSO [20] considers a *L*_1_-norm penalty and has the advantage of directly removing irrelevant predictors by shrinking their coefficients to zero. More recently, the elastic net [21] which combines the advantages of the Ridge and LASSO regressions has been proposed. In the presence of correlated variables, it outperforms LASSO by favoring the selection of sets of variables. A comparison between these methods in application to genome selection is presented in [22]. Although widely used, regression analysis is based on an ad hoc model that may not reflect the physical nature of the observed data. Further, it does not explicitly accommodate correlations between the candidate biomarkers as well as measurement errors.

The Bayesian framework offers an alternative formulation of model selection. The candidate models are assigned prior probabilities that are combined with the likelihood function to yield the so-called posterior probability. The latter summarizes all the available information to make the decision. In this context, deciding in favor of the a posteriori most probable model is optimal in the sense that it minimizes the risk associated to the 0/1 cost-function. There have been a lot of debates over the use of Bayesian techniques in place of frequentist approaches, but they do not address exactly the same question. Frequentist methods are designed to test the departure of the data from a pre-defined null hypothesis. In contrast, Bayesian selection procedures evaluate the plausibility of a given hypothesis given a set of candidate hypotheses hence are conveniently well-suited to multiple hypotheses testing. Thus, non-nested models can be compared in a straightforward manner. Another fundamental difference is the treatment of unknown model parameters. In the frequentist approach, they are classically replaced by estimates whereas in the Bayesian formulation, they are integrated. The latter procedure has the advantage of automatically penalizing complex models, as discussed in [23], but often leads to intractable calculations. An additional advantage is that correlations between the model variables can be easily accounted for in the design of the prior distributions. As for the integration over the unknown parameters, several solutions have been developed. The Laplace approximation of the integrand leads to the well-known Bayesian information criterion (BIC). As an alternative, numerical integrations can be performed based on stochastic sampling techniques such as Markov Chain Monte Carlo (MCMC) methods [24]. Either across or within model-based techniques can be considered. In the first case, the model index is sampled jointly with the parameters conditionally upon the observations. A well-known algorithm is the Reversible-Jump MCMC but moves between the different parameter spaces are difficult to design. In the second case, posterior samples of the parameters are generated conditionally upon each candidate model and then used to evaluate the integrated likelihood, also called evidence [25]. Nevertheless, the harmonic mean-based estimator exhibits instabilities [26]. Applications of the MCMC Bayesian model selection methods in genomics can be found in [27, 28].

In this paper, a Bayesian setting is adopted to identify a set of protein biomarkers from experimental data consisting of measured protein concentrations and the associated biological statuses of a population of individuals. The novelty is that the decision is not made protein by protein. As an alternative, the problem is formulated as directly finding the best partition of the list of proteins into two subsets, namely discriminant and non-discriminant, in the sense that it yields the highest posterior probability. Regardless of their discriminative power, the proteins are assumed Gaussian distributed. However, for the subset of biomarkers, the parameters of the Gaussian distribution take different values depending on the biological status whereas this is not the case for the second subset of proteins. The preliminary version of this hierarchical model has been presented in [29]. Its advantages are threefold. First, it is not based on an ad hoc explanatory model unlike regression analysis. Second, the proteins within a given group are assumed a priori correlated and the dependency structure is integrated out along with the remainder of the unknown model parameters so that only the protein partition is estimated. Thus, our approach intrinsically takes into account correlations between the candidate biomarkers. Third, by choosing appropriate conjugate prior distributions for the parameters, the model evidences can be calculated in closed form and there is no need to resort to computationally extensive numerical techniques. Finally, we show that our hierarchical model can be easily extended to address errors in the measured concentrations.

## Problem formulation, proposed models, and methods

To formulate the biomarker selection problem and construct its solution in the proposed framework, we first introduce the basic modeling for the relevant quantities/variables at hand: the biological status, protein concentrations, number of individuals,…including the descriptions of the considered observation models.

**Distribution for status and concentration** Regarding the biological status, it is denoted by *b* and takes two values, $\mathcal {H}$ and ${\mathcal {P}}$, for healthy and pathological. It is conveniently described by a Bernoulli random variable *B* with parameter *p*

1 $$ B | p \sim {\mathcal{B}} (b \,;\, p) \,.  $$

Regarding the proteins, let us note *P* is their number and $\boldsymbol {x}\in \mathbb {R}^{P}$ is the collection of their concentrations. Each protein can be discriminant or non-discriminant and then accordingly labeled by + or −. The vector ***x*** ^+^ and ***x*** ^−^, with sizes *P* ^+^ and *P* ^−^ (we have *P*=*P* ^+^+*P* ^−^), stand for the respective concentrations. Otherwise, within the *P* proteins, there are 2^*P*^ possible partitions referred to as ***δ***∈{+,−}^*P*^ since each protein can be discriminant and non-discriminant. Following the clinically observed behavior, from a probabilistic standpoint, the concentrations are described by normal distributions in order to account for biological variabilities within the populations. Specifically, for the non-discriminant proteins, the concentration vector ***x*** ^−^ is modeled by a unique multivariate normal distribution with common parameters $\left (\boldsymbol {m}_{\mathcal {C}},\boldsymbol {\Gamma }_{\mathcal {C}}\right)$ regardless the biological status:

2 $$\begin{array}{*{20}l} \boldsymbol{X}^{-} | \boldsymbol{m}_{\mathcal{C}},\boldsymbol{\Gamma}_{\mathcal{C}} \sim \mathcal{N}(\boldsymbol{x}^{-}; \boldsymbol{m}_{\mathcal{C}},\boldsymbol{\Gamma}_{\mathcal{C}}) \,. \end{array} $$

On the other hand, for discriminant ones, the concentration vector ***x*** ^+^ is modeled, conditionally on status *b*, by a multivariate normal distribution with mean and precision $\left (\boldsymbol {m}_{\mathcal {H}}, \boldsymbol {\Gamma }_{\mathcal {H}}\right)$ and $(\boldsymbol {m}_{\mathcal {P}}, \boldsymbol {\Gamma }_{\mathcal {P}})$ for healthy and pathological, respectively:

3 $$ \begin{cases} \boldsymbol{X}^{+} | b={\mathcal{P}},\boldsymbol{\delta}, \boldsymbol{m}_{\mathcal{P}}, \boldsymbol{\Gamma}_{\mathcal{P}} &\sim \mathcal{N}\left({\boldsymbol{x}^{+} \,;\, \boldsymbol{m}_{\mathcal{P}}, \boldsymbol{\Gamma}_{\mathcal{P}}}\right) \,, \\ \boldsymbol{X}^{+} | b=\mathcal{H},\boldsymbol{\delta}, \boldsymbol{m}_{\mathcal{H}}, \boldsymbol{\Gamma}_{\mathcal{H}} &\sim \mathcal{N}\left({\boldsymbol{x}^{+} \,;\, \boldsymbol{m}_{\mathcal{H}}, \boldsymbol{\Gamma}_{\mathcal{H}}}\right) \,. \\ \end{cases}  $$

Marginally, the concentrations of discriminant proteins are distributed according to a mixture of two Gaussian distributions

$$\begin{array}{*{20}l} &\boldsymbol{X}^{+} | p, \boldsymbol{m}_{\mathcal{P}}, \boldsymbol{\Gamma}_{\mathcal{P}}, \boldsymbol{m}_{\mathcal{H}}, \boldsymbol{\Gamma}_{\mathcal{H}} \sim p\, \mathcal{N}\left(\boldsymbol{x}^{+}; \boldsymbol{m}_{\mathcal{H}}, \boldsymbol{\Gamma}_{\mathcal{H}}\right)\\ &\quad+ (1-p) \mathcal{N}\left(\boldsymbol{x}^{+}; \boldsymbol{m}_{\mathcal{P}}, \boldsymbol{\Gamma}_{\mathcal{P}}\right) \,. \end{array} $$

In addition, it is assumed that ***X*** ^+^ and ***X*** ^−^ are conditionally independent.

The parameters of the distributions are collected in the vector $\boldsymbol {\theta } = \left [\boldsymbol {m}_{\mathcal {P}}, \boldsymbol {\Gamma }_{\mathcal {P}}, \boldsymbol {m}_{\mathcal {H}}, \boldsymbol {\Gamma }_{\mathcal {H}}, \boldsymbol {m}_{\mathcal {C}}, \boldsymbol {\Gamma }_{\mathcal {C}}, p\right ]$ considered as unknown. It is important to keep in mind that the quantity of interest is the partition ***δ***.

**Distribution for the individuals** The total number of individual is *N* and (***x***_*n*_,*b*_*n*_) is the *n*th concentration vector and status. They are modeled as independent conditionally on ***θ***. Let us denote $\boldsymbol {\underline {x}}$ (size *P*×*N*) as the matrix of concentrations and ***b*** (size *N*) as the vector of biological statuses. Also, let $\mathcal {I}_{\mathcal {H}}$ and $\mathcal {I}_{\mathcal {P}}$ be the subsets of indices for healthy and pathological individuals, respectively, and $N_{\mathcal {H}}, N_{\mathcal {P}}$ their cardinality. For notational convenience, we introduce: $N_{\mathcal {C}}=N_{\mathcal {H}} + N_{\mathcal {P}}$ and $\mathcal {I}_{\mathcal {C}} = \mathcal {I}_{\mathcal {P}} \cup \mathcal {I}_{\mathcal {H}}$ (where $N_{\mathcal {C}}=N$ and $\mathcal {I}_{\mathcal {C}}=\{1,2,\dots,N\}$).

Given the models (1) for the status and (2)–(3) for the non-discriminant and the discriminant proteins, based on the assumptions that $\boldsymbol {X}^{+}_{n}$ and $\boldsymbol {X}^{-}_{n}$ are conditionally uncorrelated and that the individual concentrations are also conditionally independent, the distribution of the concentrations and status $(\boldsymbol {\underline {x}},\boldsymbol {b})$, given the unknown parameters ***θ*** and partition ***δ***, is:

4 $$\begin{array}{*{20}l} f_{\boldsymbol{\underline{X}},\boldsymbol{B}\vert \boldsymbol{\Theta}, \boldsymbol{\Delta}} & (\boldsymbol{\underline{x}},\boldsymbol{b} \vert \boldsymbol{\theta}, \boldsymbol{\delta})\\ = & \prod_{n} f_{\boldsymbol{X}, B\vert \boldsymbol{\Theta}, \boldsymbol{\Delta}}\left(\boldsymbol{x}_{n},b_{n} \vert \boldsymbol{\theta}, \boldsymbol{\delta}\right)\\ = & \prod_{n} f_{\boldsymbol{X} \vert B, \boldsymbol{\Theta}, \boldsymbol{\Delta}}\left(\boldsymbol{x}_{n} \vert b_{n},\boldsymbol{\theta}, \boldsymbol{\delta}\right) ~ \mathbb{P}_{B\vert\boldsymbol{\Theta}} \left(b_{n} \vert \boldsymbol{\theta}\right) \\ = & \prod_{n \in \mathcal{I}_{\mathcal{P}}}\mathcal{N}\left(\boldsymbol{x}_{n}^{+};\boldsymbol{m}_{\mathcal{P}},\boldsymbol{\Gamma}_{\mathcal{P}}\right)\\ &\times\prod_{n \in \mathcal{I}_{\mathcal{H}}}\mathcal{N}\left(\boldsymbol{x}_{n}^{+};\boldsymbol{m}_{\mathcal{H}},\boldsymbol{\Gamma}_{\mathcal{H}}\right) \prod_{n \in \mathcal{I}_{\mathcal{C}}}\mathcal{N}\left(\boldsymbol{x}_{n}^{-};\boldsymbol{m}_{\mathcal{C}},\boldsymbol{\Gamma}_{\mathcal{C}}\right)\\ &\times\prod_{n \in \mathcal{I}_{\mathcal{C}}} \mathbb{P}_{B\vert\boldsymbol{\Theta}} (b_{n} \vert \boldsymbol{\theta})  \end{array} $$

It can be seen (see Appendix 1) that the exponential arguments of the Gaussian distributions in the first three factors can be reformulated based on the empirical means and covariances for each index set $\mathcal {I}_{\mathcal {P}}$, $\mathcal {I}_{\mathcal {H}}$, and $\mathcal {I}_{\mathcal {C}}$. The result is shown here only for the first factor:

5 $$\begin{array}{*{20}l} \prod_{n\in\mathcal{I}_{\mathcal{P}}} & \mathcal{N}\left(\boldsymbol{x}_{n}^{+}; {\boldsymbol{m}}_{\mathcal{P}},{\boldsymbol{\Gamma}}_{\mathcal{P}}\right)= (2\pi)^{-P^{+} N_{\mathcal{P}}/2} \vert {\boldsymbol{\Gamma}}_{\mathcal{P}}\vert^{N/2}\\ & \exp\left[\!-\frac{N_{\mathcal{P}}}{2}\text{Tr}\left({\boldsymbol{\Gamma}}_{\mathcal{P}} \left[\!\bar{\mathbf{R}}_{\mathcal{P}}^{+} + \left(\bar{\boldsymbol{x}}_{\mathcal{P}}^{+} -{\boldsymbol{m}}_{\mathcal{P}}\right)\left(\bar{\boldsymbol{x}}_{\mathcal{P}}^{+} -{\boldsymbol{m}}_{\mathcal{P}} \right)^{\mathrm{t}} \!\right] \right)\!\right]  \end{array} $$

where $\bar {\boldsymbol {x}}^{+}_{\mathcal {P}}$ and $\bar {\mathbf {R}}_{\mathcal {P}}^{+}$ are the empirical mean and covariance of the $\boldsymbol {x}_{n}^{+}$ for the individuals $n\in \mathcal {I}_{\mathcal {P}}$. Moreover, regarding the probability for the statuses, we have:

$$\prod\limits_{n \in \mathcal{I}_{\mathcal{C}}} \mathbb{P}_{B\vert\boldsymbol{\Theta}} (b_{n} \vert \boldsymbol{\theta}) = p^{N_{\mathcal{P}}}(1-p)^{N_{\mathcal{H}}} $$

that is only based on the size of each index set $\mathcal {I}_{\mathcal {P}}$ and $\mathcal {I}_{\mathcal {H}}$.

**Observations** Given the previously described concentrations, the proposed developments include two cases for the observation model:

1. In the first one, the concentrations ***x***_*n*_ are directly observed.
2. The second one accounts for noise: observations write ***y***_*n*_=***x***_*n*_+***ε***_*n*_, where ***ε***_*n*_ is modeled as a zero-mean Gaussian vector with precision ***Γ***_*ε*_.

Both of them account for biological variabilities and the latter also includes technological variabilities that arise from both the functioning of the measurement system itself and the post-processing of the spectra. These models are referred to as “noiseless model” and “noisy model”. The corresponding variable selection methods are respectively presented in Sections 3.1 and 3.2.

**Prior distributions** The choice of the prior distribution for the unknown parameters is important. First of all, it must allow us to account for available information (e.g., nominal values and uncertainties, strong uncertainties,…). Second, it should also enable analytical calculations or numerical computations. To this end, the prior density is chosen as a separable prior distribution, i.e., for the noiseless case

6 $$\begin{array}{*{20}l} \pi_{\boldsymbol{\Theta}\vert \boldsymbol{\Delta}}(\boldsymbol{\theta}\vert \boldsymbol{\delta}) = &\pi_{\mathcal{P}}\left(\boldsymbol{m}_{\mathcal{P}},\boldsymbol{\Gamma}_{\mathcal{P}}\vert \boldsymbol{\delta}\right) ~ \pi_{\mathcal{H}}\left(\boldsymbol{m}_{\mathcal{H}},\boldsymbol{\Gamma}_{\mathcal{H}}\vert \boldsymbol{\delta}\right)\\ & \pi_{\mathcal{C}}\left(\boldsymbol{m}_{\mathcal{C}},\boldsymbol{\Gamma}_{\mathcal{C}}\vert \boldsymbol{\delta}\right) ~ \pi_{P}(p) \end{array} $$

for $\left (\boldsymbol {m}_{\mathcal {P}},\boldsymbol {\Gamma }_{\mathcal {P}}\right)$, $\left (\boldsymbol {m}_{\mathcal {H}},\boldsymbol {\Gamma }_{\mathcal {H}}\right)$, $(\boldsymbol {m}_{\mathcal {C}},\boldsymbol {\Gamma }_{\mathcal {C}})$, and *p*. For the noisy case, in addition, we have a factor *π*(***Γ***_*ε*_) for ***Γ***_*ε*_ independent from the other variables. Regarding these five variables individually, the choice is driven by a conjugation principle [30]:

- The probability *p* is assumed a Beta distributed variable with parameter (*a*,*b*).
- The (***m***_×_,***Γ***_×_) are assumed to be Normal-Wishart $\mathcal {NW}$ distributed with parameters (***μ***_×_,*η*_×_,***Λ***_×_,*ν*_×_), for $\times \in \{{\mathcal {P}},{\mathcal {H}},\mathcal {C}\}$. See Appendix 2.
- The precision ***Γ***_*ε*_ is under a Wishart distribution with parameters (***Λ***_*ε*_,*ν*_*ε*_). See Appendix 2.

In the subsequent developments, we proceed with the calculation of the posterior probability for the partitions ***δ*** in the two cases: noiseless concentrations in Section 3.1 and noisy concentrations in Section 3.2. One of the novelty is an explicit analytical result for the noiseless case and a precise approximation for the noisy case.

### Selection using the noiseless data

**Optimal decision-maker** The question of the paper is the one of the identification of a set of discriminant proteins, and it amounts to making a decision regarding the partition ***δ***. To build an optimal decision-maker, a binary loss is considered that assigns a null loss to the correct decision and a unitary loss to the incorrect decisions. The risk is the mean loss over the models ***δ***, the data ($\boldsymbol {\underline {x}},\boldsymbol {b}$), and the unknown parameters ***θ***. The optimal decision-maker is defined as the risk minimizer, and it is known to be the one that selects the most a posteriori probable model. It should be noted that alternative loss functions could be chosen, for instance, one that would penalize differently erroneous partitions depending on the number of biomarkers properly identified. In this case, the decision would still be based on the posterior probabilities but with a different rule. However, our choice not only leads to a simple identification procedure but also naturally prevents overfitting.

Thus, the point is to calculate the posterior probability $\mathbb {P}_{\boldsymbol {\Delta }\vert \boldsymbol {\underline {X}},\boldsymbol {B}} (\boldsymbol {\delta }\vert \boldsymbol {\underline {x}},\boldsymbol {b})$ for each candidate model ***δ***. It is carried out using the Bayes rule as:

7 $$  \mathbb{P}_{\boldsymbol{\Delta}\vert\boldsymbol{\underline{X}},\boldsymbol{B}} (\boldsymbol{\delta}\vert \boldsymbol{\underline{x}},\boldsymbol{b}) = \frac{\mathbb{P}(\boldsymbol{\Delta}=\boldsymbol{\delta}) ~ f_{\boldsymbol{\underline{X}},\boldsymbol{B}\vert \boldsymbol{\Delta}}(\boldsymbol{\underline{x}},\boldsymbol{b} \vert \boldsymbol{\delta}) } { \sum_{\boldsymbol{\delta}} \mathbb{P}(\boldsymbol{\Delta}=\boldsymbol{\delta}) ~ f_{\boldsymbol{\underline{X}},\boldsymbol{B}\vert \boldsymbol{\Delta}}(\boldsymbol{\underline{x}},\boldsymbol{b} \vert \boldsymbol{\delta}) }  $$

and it crucially depends on the so-called evidence

$$\begin{array}{*{20}l} f_{\boldsymbol{\underline{X}},\boldsymbol{B}\vert \boldsymbol{\Delta}}(\boldsymbol{\underline{x}},\boldsymbol{b} \vert \boldsymbol{\delta}) & = \int_{\boldsymbol{\theta}} f_{\boldsymbol{\underline{X}},\boldsymbol{B}, \boldsymbol{\Theta} \vert \boldsymbol{\Delta}}(\boldsymbol{\underline{x}},\boldsymbol{b},\boldsymbol{\theta}\vert \boldsymbol{\delta}) ~\mathrm{d}\boldsymbol{\theta} \end{array} $$

which can be rewritten by factorization

8 $$\begin{array}{*{20}l}{} f_{\boldsymbol{\underline{X}},\boldsymbol{B}\vert \boldsymbol{\Delta}}(\boldsymbol{\underline{x}},\boldsymbol{b} \vert \boldsymbol{\delta}) & = \int_{\boldsymbol{\theta}} \pi_{\boldsymbol{\Theta}\vert \boldsymbol{\Delta}}(\boldsymbol{\theta}\vert \boldsymbol{\delta}) ~ f_{\boldsymbol{\underline{X}},\boldsymbol{B}\vert \boldsymbol{\Theta}, \boldsymbol{\Delta}}(\boldsymbol{\underline{x}},\boldsymbol{b} \vert \boldsymbol{\theta}, \boldsymbol{\delta}) ~\mathrm{d}\boldsymbol{\theta} \,. \end{array} $$

This calculation is the main difficulty of the paper and more generally in variable and model selection.

In order to carry out this calculation, let us note that the likelihood $f_{\boldsymbol {\underline {X}},\boldsymbol {B}\vert \boldsymbol {\Theta }, \boldsymbol {\Delta }}(\boldsymbol {\underline {x}},\boldsymbol {b} \vert \boldsymbol {\theta }, \boldsymbol {\delta })$ factorizes (see Eq. (4)) and that the prior *π*_***Θ***|***Δ***_(***θ***|***δ***) also factorizes (see Eq. (6)). So, we have:

$${\begin{aligned} f_{\boldsymbol{\underline{X}},\boldsymbol{B}\vert \boldsymbol{\Delta}}(\boldsymbol{\underline{x}},\boldsymbol{b} \vert \boldsymbol{\delta}) &= \int_{\boldsymbol{\theta}} ~\mathcal{NW}(\boldsymbol{m}_{\mathcal{P}},\boldsymbol{\Gamma}_{\mathcal{P}})~ \mathcal{NW}(\boldsymbol{m}_{\mathcal{H}},\boldsymbol{\Gamma}_{\mathcal{H}})\\&~~ \mathcal{NW}(\boldsymbol{m}_{\mathcal{C}},\boldsymbol{\Gamma}_{\mathcal{C}}) ~ \pi_{P}(p) \\ & \prod_{n \in \mathcal{I}_{\mathcal{P}}}\mathcal{N}\left(\boldsymbol{x}_{n}^{+};\boldsymbol{m}_{\mathcal{P}},\boldsymbol{\Gamma}_{\mathcal{P}}\right) \prod_{n \in \mathcal{I}_{\mathcal{H}}}\mathcal{N}\left(\boldsymbol{x}_{n}^{+};\boldsymbol{m}_{\mathcal{H}},\boldsymbol{\Gamma}_{\mathcal{H}}\right)\\ &\prod_{n \in \mathcal{I}_{\mathcal{C}}}\mathcal{N}\left(\boldsymbol{x}_{n}^{-};\boldsymbol{m}_{\mathcal{C}},\boldsymbol{\Gamma}_{\mathcal{C}}\right) \prod_{n \in \mathcal{I}_{\mathcal{C}}} \mathbb{P}_{B\vert\boldsymbol{\Theta}} (b_{n} \vert \boldsymbol{\theta})~\mathrm{d}\boldsymbol{\theta} \end{aligned}} $$

that can itself be factorized in four integrals: three w.r.t. the couple of variable regarding the concentrations $(\boldsymbol {m}_{\mathcal {P}},\boldsymbol {\Gamma }_{\mathcal {P}})$, $(\boldsymbol {m}_{\mathcal {H}},\boldsymbol {\Gamma }_{\mathcal {H}})$, $(\boldsymbol {m}_{\mathcal {C}},\boldsymbol {\Gamma }_{\mathcal {C}})$ and one w.r.t. the prevalence *p*:

$$f_{\boldsymbol{\underline{X}},\boldsymbol{B}\vert \boldsymbol{\Delta}}(\boldsymbol{\underline{x}},\boldsymbol{b} \vert \boldsymbol{\delta}) = \mathcal{I}_{\mathcal{P}}^{+}(\boldsymbol{\underline{x}}) ~ \mathcal{I}_{\mathcal{H}}^{+}(\boldsymbol{\underline{x}}) ~ \mathcal{I}_{\mathcal{C}}^{-}(\boldsymbol{\underline{x}}) ~ \mathcal{J}(\boldsymbol{b}) $$

where the integrals w.r.t. the ***m***_×_,***Γ***_×_ read

9 $$  \begin{aligned} \mathcal{I}_{\times}^{\star}(\boldsymbol{\underline{x}}) &= \int_{\boldsymbol{m}_{\times}, \boldsymbol{\Gamma}_{\times}} \mathcal{NW}(\boldsymbol{m}_{\times},\boldsymbol{\Gamma}_{\times}) \, \prod_{n\in \mathcal{I}_{\times}} \mathcal{N}(\boldsymbol{x}_{n}^{\star}; \boldsymbol{m}_{\times}, \Gamma_{\times})\\&\quad~\mathrm{d}\boldsymbol{m}_{\times} \, \mathrm{d}\boldsymbol{\Gamma}_{\times} \,, \end{aligned}  $$

with $\times \in \{{\mathcal {P}},{\mathcal {H}},\mathcal {C}\}$ and ⋆∈{+,−} and the integral w.r.t. *p* reads

$$\mathcal{J}(\boldsymbol{b}) = \int_{p} \pi_{P}(p) \, \prod_{n \in \mathcal{I}_{\mathcal{C}}} \mathbb{P}_{\boldsymbol{B}\vert\boldsymbol{\Theta}} (b_{n} \vert \boldsymbol{\theta}) \, \mathrm{d} p \,. $$

As far as the three integrals $\mathcal {I}_{\times }^{\star }$ are concerned, the calculations are founded on the reduced form (5) for the likelihood (including empirical means and variances) and on the Normal-Wishart prior (23) in Appendix 2 for the (***m***_×_,***Γ***_×_). Practically, thanks to the conjugacy property, the integrand in (9) involves the posterior for (***m***_×_,***Γ***_×_) which is Normal-Wishart with parameters

$$\begin{array}{*{20}l} \nu^{\star\text{pst}}_{\times} =~& \nu_{\times} +N_{\times}\\ \eta_{\times}^{\star\text{pst}} =~& \eta_{\times} +N_{\times} \\ \boldsymbol{\mu}_{\times}^{\star\text{pst}} =~& {\left(N_{\times}\bar{\boldsymbol{x}}_{\times}^{\star}+ \eta_{\times} \boldsymbol{\mu}_{\times} \right)}/{(N_{\times}+\eta_{\times})}\\ \left(\boldsymbol{\Lambda}_{\times}^{\star\text{pst}}\right)^{-1} =~& \left(\boldsymbol{\Lambda}_{\times}\right)^{-1}+ N_{\times}\bar{\mathbf{R}}_{\times}^{\star}+ N_{\times}\eta_{\times} \left(\boldsymbol{\mu}_{\times}-\bar{\boldsymbol{x}}^{\star}_{\times}\right)\\&\times\left(\boldsymbol{\mu}_{\times}-\bar{\boldsymbol{x}}^{\star}_{\times}\right)^{\mathrm{t}} / \left({N_{\times}+\eta_{\times}}\right)  \end{array} $$

where “ pst” stands for posterior. The finalization of the development relies on the fact that the Normal-Wishart density sums to one: without effective complicate calculus, this yields the result as the ratio of normaliation constants

$$\mathcal{I}_{\times}^{\star} = \frac {\mathcal{KNW}^{\star\text{pst}}_{\times}}{\mathcal{KNW}^{\star\text{pri}}_{\times}} $$

where $\mathcal {KNW}$ is the normalizing constant of the Normal-Wishart density given by (24) in Appendix 2 and where “ pri/ pst” stands for “prior/posterior”.

As a whole, the analytical calculation of the integral in (8) is possible and yields:

10 $${} f_{\boldsymbol{\underline{X}},\boldsymbol{B}\vert \boldsymbol{\Delta}}(\boldsymbol{\underline{x}},\boldsymbol{b} \vert \boldsymbol{\delta}) \propto \frac {\mathcal{KNW}^{+\text{pst}}_{\mathcal{P}}}{\mathcal{KNW}^{+\text{pri}}_{\mathcal{P}}} \frac {\mathcal{KNW}^{+\text{pst}}_{\mathcal{H}}}{\mathcal{KNW}^{+\text{pri}}_{\mathcal{H}}} \frac {\mathcal{KNW}^{-\text{pst}}_{\mathcal{C}}}{\mathcal{KNW}^{-\text{pri}}_{\mathcal{C}}}  $$

rendering the usually complex calculations of the evidences straightforward.

Assuming that all candidate models are equally a priori probable, from Eq. (7), the posterior probability across the 2^*P*^ models can be inferred. The selected model is the one which maximizes this probability. It should be noted that if prior information is available such as protein-to-protein interactions (PPI’s), it can be taken into account by assigning a higher probability to partitions wherein the related proteins are in the same subset (either discriminant or not). In Eq. (10), the normalizing constants for the posterior distributions depend on the empirical covariance matrices of the population of individuals for the discriminant proteins and the non-discriminant ones, respectively. Their computation is expensive. However, it suffices to compute once the full covariance matrix for all the proteins and then remove the appropriate raws and columns for the 2^*P*^ configurations to be tested.

### Selection using noisy data

The model presented above assumes that the concentrations are directly observed. Although this assumption leads to closed-form expressions of the posterior probabilities, it may be too simplifying. In practice, the concentrations are known up to an uncertainty and this section extends the above-detailed developments to account for these uncertainties. However, this comes at the price of intractable calculations, and to overcome this difficulty, we propose a suitable approximation. As introduced above, the measured concentrations are modeled as ***y***_*n*_=***x***_*n*_+***ε***_*n*_ where ***ε***_*n*_ is a zero-mean Gaussian vector with precision ***Γ***_*ε*_, therefore $\boldsymbol {y}_{n}|\boldsymbol {x}_{n} \sim \mathcal {N}(\boldsymbol {x}_{n}; \mathbf {0},\boldsymbol {\Gamma }_{\varepsilon })$. Similarly to the previous section, the vectors of observed concentrations are stacked in a matrix $\boldsymbol {\underline {y}}$ of dimension *P*×*N*. To select the most probable model, the evidence $f_{\boldsymbol {\underline {Y}},\boldsymbol {B}\vert \boldsymbol {\Delta }}(\boldsymbol {\underline {y}},\boldsymbol {b} \vert \boldsymbol {\delta })$ must be calculated for each candidate model (it was $f_{\boldsymbol {\underline {X}},\boldsymbol {B}\vert \boldsymbol {\Delta }}(\boldsymbol {\underline {x}},\boldsymbol {b} \vert \boldsymbol {\delta })$ for the noiseless model). The difficulty is that the calculation of evidence requires not only the marginalization of the model parameters but also of the true concentrations. Furthermore, the precision ***Γ***_*ε*_ is assumed unknown and must also be marginalized. For notational convenience, we state: $\tilde {\boldsymbol {\theta }} = \left [\boldsymbol {\theta }, \boldsymbol {\Gamma }_{\varepsilon } \right ]$ as an extended vector of unknown parameters.

By taking into account the conditional independencies, the evidence can be expressed as:

11 $$ {}\begin{aligned} f_{\boldsymbol{\underline{Y}},\boldsymbol{B}\vert \boldsymbol{\Delta}}(\boldsymbol{\underline{y}},\boldsymbol{b} \vert \boldsymbol{\delta}) &= \int_{\boldsymbol{\underline{x}}}\int_{\tilde{\boldsymbol{\theta}}} \prod_{n\in \mathcal{I}_{\mathcal{C}}} \mathcal{N}(\boldsymbol{y}_{n}; \boldsymbol{x}_{n},\boldsymbol{\Gamma}_{\varepsilon})\\ &\quad\prod_{n\in \mathcal{I}_{\mathcal{P}}} \mathcal{N}\left(\boldsymbol{x}_{n}^{+}; \boldsymbol{m}_{\mathcal{P}},\boldsymbol{\Gamma}_{\mathcal{P}}\right) \prod_{n\in \mathcal{I}_{\mathcal{H}}} \mathcal{N} \left(\boldsymbol{x}_{n}^{+}; \boldsymbol{m}_{\mathcal{H}}, \boldsymbol{\Gamma}_{\mathcal{H}}\right)\\ &\quad~\prod_{n\in \mathcal{I}_{\mathcal{C}}} \mathcal{N} \left(\boldsymbol{x}_{n}^{-}; \boldsymbol{m}_{\mathcal{C}}, \Gamma_{\mathcal{C}}\right) ~\prod_{n \in \mathcal{I}_{\mathcal{C}}} \mathbb{P}_{B\vert\boldsymbol{\Theta}} (b_{n} \vert \boldsymbol{\theta})\\&\quad ~\pi_{\tilde{\boldsymbol{\Theta}}}\left(\tilde{\boldsymbol{\theta}}\vert\boldsymbol{\delta}\right) ~ \mathrm{d}\tilde{\boldsymbol{\theta}} ~ \mathrm{d}\boldsymbol{\underline{x}} \end{aligned}  $$

This multiple integral can be handled in several manners. We propose to first perform integration with respect to $\tilde {\boldsymbol {\theta }}$ and then to integrate the result with respect to $\boldsymbol {\underline {x}}$; hence, Eq. (11) can be rewritten as:

$$f_{\boldsymbol{\underline{Y}},\boldsymbol{B}\vert \boldsymbol{\Delta}}(\boldsymbol{\underline{y}},\boldsymbol{b} \vert \boldsymbol{\delta}) = \int_{\boldsymbol{\underline{x}}} \mathcal{I}_{\varepsilon}(\boldsymbol{\underline{x}}) \, \mathcal{I}_{\mathcal{P}}^{+}(\boldsymbol{\underline{x}}) \, \mathcal{I}_{\mathcal{H}}^{+}(\boldsymbol{\underline{x}}) \, \mathcal{I}_{\mathcal{C}}^{-}(\boldsymbol{\underline{x}}) \, \mathrm{d}\boldsymbol{\underline{x}} $$

where

12 $$ \mathcal{I}_{\varepsilon}(\boldsymbol{\underline{x}}) = \int_{\boldsymbol{\Gamma}_{\varepsilon}} \, \mathcal{W}(\boldsymbol{\Gamma}_{\varepsilon}) \, \prod_{n\in \mathcal{I}_{\mathcal{C}}} \mathcal{N}(\boldsymbol{y}_{n}; \boldsymbol{x}_{n}, \boldsymbol{\Gamma}_{\varepsilon}) \, \mathrm{d}\boldsymbol{\Gamma}_{\varepsilon}  $$

and in the same manner as previously

13 $$ \begin{aligned} \mathcal{I}_{\times}^{\star}(\boldsymbol{\underline{x}}) &= \int_{\boldsymbol{m}_{\times}, \boldsymbol{\Gamma}_{\times}} ~ \mathcal{NW}(\boldsymbol{m}_{\times},\boldsymbol{\Gamma}_{\times}) \, \prod_{n\in \mathcal{I}_{\times}} \mathcal{N} \left(\boldsymbol{x}_{n}^{\star}; \boldsymbol{m}_{\times}, \Gamma_{\times}\right)\\& \mathrm{d}\boldsymbol{m}_{\times} \, \mathrm{d}\boldsymbol{\Gamma}_{\times} \end{aligned}  $$

with $\times \in \{{\mathcal {P}},{\mathcal {H}},\mathcal {C}\}$ and ⋆∈{+,−}. The integrals (12) and (13) can be calculated analytically.

On the one hand, the integrand of (12) can be rewritten, up to a proportionality constant, as the distribution of the precision matrix ***Γ***_*ε*_ conditionally upon $\boldsymbol {\underline {y}}$ and $\boldsymbol {\underline {x}}$. This distribution is Wishart with parameters $\left (\nu _{\varepsilon }^{\text {pst}}, \boldsymbol {\Lambda }_{\varepsilon }^{\text {pst}} \right)$ expressed as:

$$\begin{array}{@{}rcl@{}} \left\{\begin{array}{lcl} \nu_{\varepsilon}^{\text{pst}}&=& \nu_{\varepsilon}^{\text{pri}}+N_{\mathcal{C}}\\ \left(\boldsymbol{\Lambda}_{\varepsilon}^{\text{pst}}\right)^{-1} &=& \left(\boldsymbol{\Lambda}_{\varepsilon}^{\text{pri}}\right)^{-1}+\sum\limits_{n=1}^{N_{\mathcal{C}}}(\boldsymbol{y}_{n}-\boldsymbol{x}_{n})(\boldsymbol{y}_{n}-\boldsymbol{x}_{n})^{\mathrm{t}}. \end{array}\right. \end{array} $$

Then, (12) can be re-arranged as:

14 $$\begin{array}{@{}rcl@{}} \mathcal{I}_{\varepsilon}(\boldsymbol{\underline{x}}) &=&\frac{{\mathcal{KW}}_{\varepsilon}^{\text{pst}}}{{\mathcal{KW}}_{\varepsilon}^{\text{pri}}}(2\pi)^{-N_{\mathcal{C}} P/2} \\ &=&\mathcal{T}_{P,N}\left(\boldsymbol{\underline{x}}; \nu_{\varepsilon}^{\text{pri}}+1-P,\boldsymbol{\underline{y}},\left(\boldsymbol{\Lambda}_{\varepsilon}^{\text{pri}}\right)^{-1},\boldsymbol{I}_{N}\right)  \end{array} $$

where ${\mathcal {KW}}_{\varepsilon }^{\star }$ is the normalization constant of the Wishart distribution and $\mathcal {T}_{P,N}(\boldsymbol {T};q,\boldsymbol {M},\boldsymbol {\Sigma },\boldsymbol {\Omega })$ denote the matrix t-distribution of parameters *q*, ***M***, ***Σ***, and ***Ω***, for a matrix ***T*** of dimensions *P*×*N*. The expression is recalled in the “Matrix variate t-distribution” section of Appendix 2.

On the other hand, the integrals $\mathcal {I}_{\times }^{\star }$ can be computed in the same manner as in the previous section using the conjugation property for the couples (***m***_×_,***Γ***_×_). Thus, we have:

15 $$ \mathcal{I}_{\times}^{\star}(\boldsymbol{\underline{x}}) = (2\pi)^{-{N_{\times} P^{\star}}/{2}} ~~ \frac {\mathcal{KNW}^{\star\text{pst}}_{\times}}{\mathcal{KNW}^{\star\text{pri}}_{\times}}  $$

with $\mathcal {KNW}^{\star \text {pst}}_{\times }$ and $\mathcal {KNW}^{\star \text {pri}}_{\times }$ the normalization constants of the prior and posterior Normal-Wishart distributions for (***m***_×_,***Γ***_×_), respectively.

In (11), the result of the first integration with respect to $\tilde {\boldsymbol {\theta }}$ does not yield an expression that can be integrated analytically w.r.t. $\boldsymbol {\underline {x}}$. To address this issue, we propose to take advantage of the fact that a matrix variate t-distribution $\mathcal {T}_{P,N}(\boldsymbol {T};q,\boldsymbol {M},\boldsymbol {\Sigma },\boldsymbol {\Omega })$ tends to a Gaussian distribution when the degrees of freedom parameter *q* tends to infinity.

In a first step, for a high enough value of $\nu _{\varepsilon }^{\text {pri}}$, (14) can be approximated as:

$$\mathcal{I}_{\varepsilon}(\boldsymbol{\underline{x}}) \simeq \prod_{n\in\mathcal{I}_{\mathcal{C}}}\mathcal{N}\left(\boldsymbol{x}_{n};\boldsymbol{y}_{n},\boldsymbol{\Lambda}_{\varepsilon}^{\text{pri}}\left(\nu_{\varepsilon}^{\text{pri}}+1-P\right)\right). $$

In a second step, the integrals $\mathcal {I}_{\times }^{\star }$ can also be approximated by Gaussian distributions although not directly. For this purpose, we focus on the factor of (15) that depends on the true concentration vectors:

16 $$ \begin{aligned} \mathcal{I}_{\times}^{\star}(\boldsymbol{\underline{x}}) &= C_{\times}^{\star} \left|\left(\boldsymbol{\Lambda}_{\times}^{\star}\right)^{-1}+ N_{\times}\bar{\mathbf{R}}_{\times}^{\star}+ N_{\times}\eta_{\times} \left(\boldsymbol{\mu}_{\times}-\bar{\boldsymbol{x}}^{\star}_{\times}\right)\right.\\&\qquad\quad\left.\times\left(\boldsymbol{\mu}_{\times}-\bar{\boldsymbol{x}}^{\star}_{\times}\right)^{\mathrm{t}} / \left({N_{\times}+\eta_{\times}}\right) \right|^{-{\nu_{\times}^{\text{pst}}}/{2}} \end{aligned}  $$

where $C_{\times }^{\star }$ is a proportionality constant.

Contrary to (14), the expression (16) does not correspond to a standard probability density function. To make the calculations tractable, we propose to replace the empirical means of the true concentrations $\bar {\boldsymbol {x}}^{\star }_{\times }$ by their approximated values computed from the measured concentrations $\bar {\boldsymbol {y}}^{\star }_{\times }$. By developing the expression of the empirical covariance matrix in (16), it ensues:

$$\begin{array}{@{}rcl@{}} \mathcal{I}_{\times}^{\star}(\boldsymbol{\underline{x}}) &\simeq& C_{\times}^{\star} \, \left|\boldsymbol{\Pi}_{\times}^{\star} +\sum_{n\in \mathcal{I}_{\times}} \left(\boldsymbol{x}_{n}^{\star}-\bar{\boldsymbol{y}}^{\star}_{\times}\right)\left(\boldsymbol{x}_{n}^{\star}-\bar{\boldsymbol{y}}^{\star}_{\times} \right)^{\mathrm{t}}\right|^{-{\nu_{\times}^{\text{pst}}}/{2}}\\ &=&C_{\times}^{\star} \, \left|\boldsymbol{\Pi}_{\times}^{\star}\left|{~}^{-{\nu_{\times}^{\text{pst}}}/{2}} \, \right|\boldsymbol{I}_{P} + \left(\boldsymbol{\Pi}_{\times}^{\star}\right)^{-1} \left(\boldsymbol{\underline{x}}_{\times}^{\star}-\bar{\boldsymbol{\underline{y}}}_{\times}^{\star}\right)\right.\\ &&\qquad\qquad\qquad\quad\times\left. \left(\boldsymbol{\underline{x}}_{\times}^{\star}-\bar{\boldsymbol{\underline{y}}}_{\times}^{\star}\right)\right|^{-{\nu_{\times}^{\text{pst}}}/{2}},\\ &\propto& \mathcal{T}_{P,N}\left(\boldsymbol{\underline{x}}_{\times}^{\star}; \nu^{\star\text{pst}}_{\times}+1-P^{\star},\bar{\boldsymbol{\underline{y}}}_{\times}^{\star},\boldsymbol{\Pi}_{\times}^{\star},\boldsymbol{I}_{N}\right) \end{array} $$

where ${\boldsymbol {\Pi }_{\times }^{\star } = \left (\boldsymbol {\Lambda }_{\times }^{\star }\right)^{-1}+ N_{\times }\eta _{\times } \left (\boldsymbol {\mu }_{\times }-\bar {\boldsymbol {x}}^{\star }_{\times }\right)\left (\boldsymbol {\mu }_{\times }-\bar {\boldsymbol {x}}^{\star }_{\times }\right)^{\mathrm {t}} \left ({N_{\times }}\\ \right.}\\+ \left.{\eta _{\times }}\right) $ and $\bar {\boldsymbol {\underline {y}}}_{\times }^{\star }$ is a matrix of dimensions *P* ^⋆^×*N*_×_ whose columns are all equal to the empirical mean $\bar {\boldsymbol {y}}^{\star }_{\times }$. Then, provided $\nu _{\times }^{\text {pri}}$ is high enough, we can also approximate this matrix variate t-distribution by a Gaussian distribution as for (12):

$$\mathcal{I}_{\times}^{\star}(\boldsymbol{\underline{x}}) \propto \mathcal \prod_{n \in \mathcal{I}_{\times}} \mathcal{N}\left(\boldsymbol{x}_{n}^{\star}; \bar{\boldsymbol{y}}^{\star}_{\times},\boldsymbol{\Pi}_{\times}^{\star}/\left(\nu_{\times}^{\text{pri}}-P^{\star}+1\right) \right) $$

The performed approximations allow us to express the integrand of the evidence (11) as a product of Gaussian distributions for the true concentration vectors. In this case, the integration can be carried out analytically. By treating separately pathological and healthy individuals, we finally obtain the following expression of the evidence:

17 $$ { \begin{aligned} f_{\boldsymbol{\underline{Y}},\boldsymbol{B}\vert \boldsymbol{\Delta}}(\boldsymbol{\underline{y}},\boldsymbol{b} \vert \boldsymbol{\delta})\simeq &\left(\frac{\eta_{\mathcal{P}}^{+\text{pri}}\eta_{\mathcal{H}}^{+\text{pri}}}{\eta_{\mathcal{P}}^{\text{pst}}~\eta_{\mathcal{H}}^{\text{pst}}}\right)^{P^{+}}\left(\frac{\eta_{\mathcal{C}}^{+\text{pri}}}{\eta_{\mathcal{C}}^{\text{pst}}}\right)^{P^{-}}\\ &\times\left\vert \boldsymbol{\Lambda}_{\mathcal{P}}^{\text{pri}} \boldsymbol{\Pi}_{p}^{+}\right\vert^{-\nu_{\mathcal{P}}^{\text{pri}}} ~ \left\vert \boldsymbol{\Lambda}_{\mathcal{H}}^{\text{pri}} \boldsymbol{\Pi}_{\mathcal{H}}^{+}\right\vert^{-\nu_{\mathcal{H}}^{\text{pri}}}\\ &\times\left\vert \boldsymbol{\Lambda}_{\mathcal{C}}^{\text{pri}} \boldsymbol{\Pi}_{\mathcal{C}}^{+}\right\vert^{-\nu_{\mathcal{C}}^{\text{pri}}}\\ & \times \left|\boldsymbol{\Sigma}_{\mathcal{P}}\right|^{-{1}/{2}}\left|\boldsymbol{\Sigma}_{\mathcal{H}}\right|^{-{1}/{2}}\\&\exp\left(-\text{Tr}\left(\boldsymbol{\Sigma}_{\mathcal{P}}^{-1}\bar{\mathbf{R}}_{\mathcal{P}}^{y}+\boldsymbol{\Sigma}_{\mathcal{H}}^{-1}\bar{\mathbf{R}}_{\mathcal{H}}^{y}\right)/2\right) \end{aligned}}  $$

In this expression, $\bar {\mathbf {R}}_{\mathcal {P}}^{y}$ and $\bar {\mathbf {R}}_{\mathcal {H}}^{y}$ are the empirical covariance matrices of the measured concentration vectors for the pathological and healthy individuals, respectively. As for $\boldsymbol {\Sigma }_{\mathcal {H}}$ and $\boldsymbol {\Sigma }_{\mathcal {P}}$, they are defined as:

$$\begin{aligned} \boldsymbol{\Sigma}_{\times} &= \frac{\Lambda_{\varepsilon}^{-1}}{\nu_{\varepsilon}^{\text{pri}}+1-P}\\&+\text{blkdiag}\left[\frac{\boldsymbol{\Pi}_{\times}^{+}}{\nu_{\times}^{\text{pri}}+1-P^{+}},\frac{\boldsymbol{\Pi}_{\mathcal{C}}}{\nu_{\mathcal{C}}^{\text{pri}}+1-P^{-}}\right] \end{aligned} $$

with $\times \in \left \{{\mathcal {P}},{\mathcal {H}}\right \}$ and blkdiag(***A***,***B***) the block-diagonal matrix with diagonal elements ***A*** and ***B***.

## Numerical evaluation

To assess the performance of the proposed method, we have performed extensive numerical experiments using both simulated and real data. They include comparisons with other methods for biomarker identification, namely:

1. The *t* test [31], which consists in comparing the means of each protein concentrations between the two cohorts, ${\mathcal {H}}$ and ${\mathcal {P}}$. If the null hypothesis, standing for the mean equality, is rejected, then the protein is declared as a biomarker. The type I error, denoted as *α*, corresponds to the incorrect rejection of a true null hypothesis. Its value is used to set the *t* test decision threshold. In this paper, it is not necessary to adjust the type I errors to account for multivariate effects. The reason is that, for fair comparison purposes, we directly select the setting that leads to the best performance of the test regarding our criterion. This point is commented in Section 4.1 and Fig. 2.
2. The LASSO method [20], based on a linear regression model in which the explanatory variables are the protein concentrations $\boldsymbol {\underline {x}}$, while the response variables are the biological statuses ***b***. The LASSO method estimates the coefficients of the model by minimizing the sum of the squared errors, with a *L*_1_-norm penalty. Then, a protein is selected as a biomarker if the value of the coefficient corresponding to its concentration is different from zero. This method introduces a regularization parameter denoted *λ*.
3. The Bhattacharyya distance [32] is a measure of similarity between two probability distributions and by extension between two populations of individuals [32]. For two multivariate normal distributions with respective mean and covariance matrix (***μ***_1_,***Σ***_1_) and (***μ***_2_,***Σ***_2_), it is given by:
  $$\begin{aligned} D_{b}&=\frac{1}{8}(\boldsymbol{\mu}_{1}-\boldsymbol{\mu}_{2})^{T}\boldsymbol{\Sigma}^{-1}(\boldsymbol{\mu}_{1}-\boldsymbol{\mu}_{2})^{T}+\frac{1}{2}\\&\quad\log\left(\frac{\text{det}(\boldsymbol{\Sigma})}{\sqrt{\text{det}(\boldsymbol{\Sigma}_{1})\text{det}(\boldsymbol{\Sigma}_{2})}} \right) \end{aligned} $$
  with ***Σ***=(***Σ***_1_+***Σ***_2_)/2.
  In the sequel, the Bhattacharyya distance is calculated for each protein by replacing the true mean and covariance matrix by their empirical estimates. The protein is declared as discriminant if the distance is greater than a fixed threshold denoted *t*. The algorithm is referred to as Bha-distance.
4. The FOHSIC algorithm as introduced in [33]. It performs feature selection based on the Hilbert-Schmidt Independence Criterion (HSIC). The authors propose an unbiased estimator of HSIC and then, assuming the number of significant features is set a priori, use a forward procedure to select them. In our context, the significant features are the biomarkers.

In this section, we refer to the method from Section 3.1 as the Bayesian Model Selection with Analytical Solution for Noiseless Data (BMS-AS-D) method, while to the method from Section 3.2 as the Bayesian Model Selection with Analytical Solution for Noisy data (BMS-AS-N) method.

Crucial to our approach is the choice of the parameters of the Normal-Wishart densities (*ν*_×_,*η*_×_,***μ***_×_,***Λ***_×_) for $\times \in \{{\mathcal {P}},{\mathcal {H}},\mathcal {C}\}$. They are referred to as the hyperparameters since the evidence (10) depends on them. In a non-informative case, the values of these hyperparameters are chosen to be (0,0,0,*∞*), while the proportionality coefficient in (10) has an undetermined form. As an alternative, to tune the parameters, we propose to use a poorly informative prior based on expert knowledge^1^ about the corresponding variables (e.g., the values in *μ*g/ml). To this end, we take advantage of the expression of the prior mean and the covariance for (***m***_×_,***Γ***_×_) as a function of the hyperparameters:

18 $$\begin{array}{@{}rcl@{}} E(\boldsymbol{\Gamma}_{\times}) &=&\nu_{\times}\boldsymbol{\Lambda}_{\times}  \end{array} $$

19 $$\begin{array}{@{}rcl@{}} E(\boldsymbol{m}_{\times}) &=&\boldsymbol{\mu}_{\times}  \end{array} $$

20 $$\begin{array}{@{}rcl@{}} V(\boldsymbol{m}_{\times}) &=&{\boldsymbol{\Lambda}_{\times}^{-1}} ~/~ {\left[\eta_{\times}\left(\nu_{\times}-P^{\ast}-1\right)\right]} \end{array} $$

21 $$\begin{array}{@{}rcl@{}} \text{cov}\left(\Gamma_{\times}^{i,j},\Gamma_{\times}^{k,l}\right) &=& \nu_{\times}\left(\Lambda_{\times}^{il}\Lambda_{\times}^{jk}+\Lambda_{\times}^{ik}\Lambda_{\times}^{jl}\right), \end{array} $$

where the superscripts *i*,*j* denote the entry (*i*,*j*) of the matrices, *E*(·) and *V*(·) refer to the expectation and the covariance matrix of a vector, respectively, while cov(·,·) stands for the covariance between two random variables. We also recall that ∗∈{ ^+^, ^−^, ^“ "^} depending whether the discriminant/non-discriminant subsets of proteins are considered or the whole set. As a consequence, the prior parameters (*ν*_×_,*η*_×_,***μ***_×_,***Λ***_×_) can be calculated from (18) to (21) and substituted in (10). Although our choice of prior is not non-informative in the strict sense, it is vague enough so as not to impact biomarker detection. This issue is investigated in the next subsection.

Finally, to calculate (17) in the noisy case, additional hyperparameters for the Wishart probability density function of the noise precision matrix have to be tuned. They are chosen such that $E(\boldsymbol {\Gamma }_{\varepsilon }) =\nu _{\varepsilon }^{\text {pri}}\boldsymbol {\Lambda }_{\varepsilon }^{\text {pri}}$ and that the elements of the covariance matrix satisfy $\text {cov}\left (\Gamma _{\varepsilon }^{i,j},\Gamma _{\varepsilon }^{k,l}\right)= \nu _{\varepsilon }^{\text {pri}} \left (\Lambda _{\varepsilon }^{\text {pri},il}\Lambda _{\varepsilon }^{\text {pri},jk}+\Lambda _{\varepsilon }^{\text {pri},ik}\Lambda _{\varepsilon }^{\text {pri},jl}\right)$. Therefore, by accounting for real-life orders of magnitudes of ***Γ***_*ε*_, the prior parameters $\left (\nu _{\varepsilon }^{\text {pri}}, \boldsymbol {\Lambda }_{\varepsilon }^{\text {pri}}\right)$ can be calculated and substituted in the probability (10).

In the next sections, we present the results of the numerical evaluations of the proposed methods using both simulated and real data.

### Evaluation using simulated data

#### Description of the simulated data and performance index

We consider the concentrations of a list of *P* proteins for a group which comprises $N_{\mathcal {H}}$ healthy and $N_{\mathcal {P}}$ pathological individuals, respectively, with $N_{\mathcal {H}}+N_{\mathcal {P}}=N$. The possible partitions for discriminant/non-discriminant proteins thus amount to 2^*P*^ and they are referred to as true models. For each true model, *N*_r_=10^5^ data realizations are simulated, hence the total number of realizations equals *N*_r_ 2^*P*^.

On the one hand, the noiseless data comprise the biological statuses *b*_*n*_ and the actual protein concentrations ***x***_*n*_ of the *N* individuals and are generated as follows. The biological statuses are sampled from the Bernoulli distribution of parameter *p*, where *p* is assumed Beta distributed of parameters *a*=1 and *b*=1, which corresponds to a uniform distribution. The concentrations of the subset of discriminant proteins are generated from the Gaussian distributions, $\mathcal {N} \left (\boldsymbol {x}_{n}^{+}; \boldsymbol {m}_{\mathcal {H}}, \boldsymbol {\Gamma }_{\mathcal {H}}\right)$ or $\mathcal {N} \left (\boldsymbol {x}_{n}^{+}; \boldsymbol {m}_{\mathcal {P}}, \boldsymbol {\Gamma }_{\mathcal {P}}\right)$, depending on the simulated biological status. The subset of non-discriminant proteins are sampled from $\mathcal {N} \left (\boldsymbol {x}_{n}^{-}; \boldsymbol {m}_{\mathcal {C}}, \boldsymbol {\Gamma }_{\mathcal {C}}\right)$. The parameters (***m***_×_,***Γ***_×_), where $\times \in \{{\mathcal {P}},{\mathcal {H}},\mathcal {C}\}$, are distributed according to the Normal-Wishart distribution $\mathcal {NW}(\nu _{\times },\eta _{\times },\boldsymbol {\mu }_{\times },\boldsymbol {\Lambda }_{\times })$. The orders of magnitudes for (***m***_×_,***Γ***_×_) are specified as: $E(\boldsymbol {m}_{\times })=10^{3}\mathbf {1}_{P^{\ast }},\ V(\boldsymbol {m}_{\times })=10^{4}\,\mathbf {I}_{P^{\ast }},\ E(\boldsymbol {\Gamma }_{\times })=10^{3}\,\mathbf {I}_{P^{\ast }},\ V(\boldsymbol {\Gamma }_{\times })=10^{4}\,\mathbf {I}_{P^{\ast }}$, where $\mathbf {1}_{P^{\ast }}\phantom {\dot {i}\!}$ denotes a vector of size *P* ^∗^ whose elements are all equal to 1 and $\mathbf {I}_{P^{\ast }}\phantom {\dot {i}\!}$ is the identity matrix of size *P* ^∗^. The same order of magnitude is considered for healthy, pathological, and common parameters, that is to say $\times \in \lbrace {\mathcal {H}},{\mathcal {P}},\mathcal {C}\rbrace $. These a priori information are used to tune the hyperparameters as given by (18)–(21).

On the other hand, the noisy data include the biological statuses *b*_*n*_ and the observed protein concentrations ***y***_*n*_ for *n*=1,2…*N*. The protein concentrations ***x***_*n*_ are generated as in the case of the noiseless observations by using the same hyperparameter setting. As for the noise ***ε***_*n*_, it is sampled as a zero-mean multivariate Gaussian random vector with precision matrix ***Γ***_*ε*_. The latter is generated from a Wishart density with parameters $\left (\nu _{\varepsilon }^{\text {pri}}, \boldsymbol {\Lambda }_{\varepsilon }^{\text {pri}}\right)$. In order to determine these hyperparameters, the a priori information is specified as: *E*(***Γ***_*ε*_)=10^−2^ **I**_*P*_, *V*(***Γ***_*ε*_)=10^−5^ **I**_*P*_. Note here that ***Γ***_*ε*_ measures the precision (inverse variance), thus the lower ***Γ***_*ε*_ is, the stronger the noise is.

For each data set, the posterior probability is computed for all possible partitions according to (10) or (17) for the BMS-AS-D and BMS-AS-N methods, respectively. Then, the most probable partition is selected.

The performance is measured in terms of the error rate *τ*, defined as:

$$\tau (\%)=\left(\sum_{i=1}^{2^{P}}{E}_{i}/\left(N_{\mathrm{r}}\times 2^{P}\right)\right) \times 100 $$

where *E*_*i*_ is the number of realizations of the *i*th partition for which the selected model is different from the true one. It should be noted that the usual type I and type II errors apply when the biomarker identification is made protein after protein but they are not relevant here, since the proteins are addressed as a whole. Indeed, as underlined in Section 3, the proposed Bayesian formulation relies on a different paradigm than the existing methods: any wrong or correct decision regards the list of proteins as a whole (and not protein-wise). Furthermore, the proposed approach minimizes a global risk and cannot be directly related to a given false discovery rate. It ensues that *τ*, which encompasses all types of errors, appears as a suitable criterion to measure the performance.

#### Results for the noiseless model

**Fig. 1:**
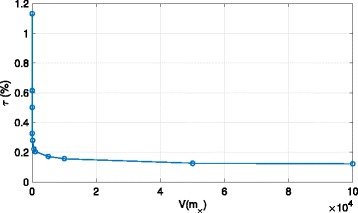
The mean is fixed, and different values of the variance are considered. *P*=3, *N*=10^3^, *N*_r_=10^5^

First of all, we investigate the sensitivity of the error rate *τ*(*%*) to the hyperparameter tuning. In Fig. 1, we plot the latter as a function of the considered variance *V*(***m***_×_) in Eqs. (18)–(21). We can observe that provided the variance is chosen superior to a given threshold, it has no impact on the biomarker detection performance. This is the case for our settings.

**Fig. 2:**
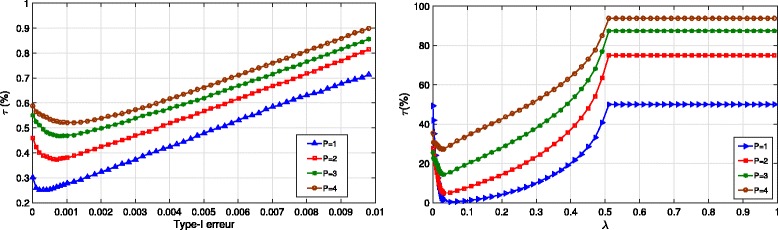
Error rate *τ*(*%*) for the *t* test with different values of the type I error (*left*) and for the LASSO with different values of *λ* (*right*)

Before going further in analyzing the performance, it should be noted that the *t* test, the LASSO, and the Battacharyya distance all require the setting of a parameter: the type I error *α*, the regularization parameter *λ*, and the threshold *t*, respectively. So as not to favor our approach, we have run all the algorithms for different values of these parameters and we have selected the best one (in order to get the lowest error rate). Such a procedure cannot be applied on real data, but it allows us to compare the proposed method to the best version of the alternative approaches. The results are given in Fig. 2 for the *t* test and the LASSO.

The performance of the BMS-AS-D method has first been evaluated as a function of the number of proteins and results are shown in Table 1. The superior performance of the BMS-AS-D method with respect to the *t* test is expected since the BMS-AS-D makes a decision jointly across all the proteins while accounting for all possible correlations between them. This issue is not addressed within the *t* test which is univariate, i.e., each protein is tested separately. The same observation can be made for the Bhattacharyya distance. Indeed, the error rate of the *t* test and the Bhattacharyya distance are very close. As for the superiority of the BMS-AS-D method over the LASSO, it is due to the fact that the latter makes the assumption of an arbitrary linear regression relationship between the biological statuses and the protein concentrations. Moreover, the difference in performance increases with the number of proteins, since the possibility of correlation increases. Indeed, in the presence of correlated variables, the number of significant variables is known to be over-estimated with the LASSO algorithm. This fact confirms the relevance of the BMS-AS-D method which can accommodate correlations between the variables.

**Table 1:** Noiseless data: *τ* (%) for different value of *P*, *N*=1000

*P*	1	2	3	4
Best *t* test	0.2555	0.4025	0.471	0.5209
Best LASSO	0.3185	4.865	14.207	27.2655
Best Bha-distance	0.2245	0.3743	0.4496	0.5008
BMS-AS-D	0.0935	0.1434	0.1563	0.1616

Regarding the number of individuals, Table 2 shows that, as expected, the performance of all the methods improves as the number of considered individuals increases. In particular, the better performance of the BMS-AS-D method is explained by the reduced variance of the protein concentration posterior distribution for a large number of individuals. Furthermore, the BMS-AS-D method outperforms the *t* test, the LASSO and the Bhattacharyya distance, regardless the number of individuals. Finally, even if the number of configurations to be tested increases exponentially with the number of proteins, the computational cost is kept reasonable owing to the analytical expression of the posterior probabilities for the different partitions.

**Table 2:** Noiseless data: *τ* (%) for different values of *N*, *P*=3

*N*	100	500	1000
*t* test	4.041	0.8938	0.471
LASSO	20.5541	15.9870	14.207
Best Bha-distance	3.6970	0.8194	0.4496
BMS-AS-D	1.71	0.32	0.1563

Last but not the least, our method can also be run for a fixed number of biomarkers as it is the case of many current feature identification algorithms such as the FOHSIC. Only the partitions with the proper number of biomarkers are studied, which amounts to assigning a null prior probability to the others. When the number of biomarkers is chosen as *M*≤*P*, the number of posterior probabilities to compute is limited to $C_{P}^{M}$ instead of 2^*P*^. Under this assumption, the performance of the BMS-AS-D is compared to that of the FOHSIC in the Tables 3, 4, 5 and 6. To run the algorithm with large *P* (≥8), the number of realizations is reduced to 10^3^.

**Table 3:** *τ* (%) *N*=1000 and *P*=3

Number of biomarkers	2
BMS-AS-D	0.0723
FOHSIC	0.2907

**Table 4:** Execution time for one simulation *N*=1000 and *P*=3

Number of biomarkers	2
BMS-AS-D	0.202 s
FOHSIC	0.732 s

**Table 5:** *τ* (%) for *P*=8, *N*_r_=10^3^, and *N*=500

*M*:	4	6
BMS-AS-D	0.3014	0.2107
FOHSIC	0.8271	0.9107

**Table 6:** *τ* (%) *M*=4, *N*_r_=10^3^, and *N*=500

*P*	8	12
BMS-AS-D	0.3014	0.1818
FOHSIC	0.8271	0.8103

As shown in Tables 3, 5, and 6, the BMS-AS-D algorithm outperforms the FOHSIC one. This is explained by the fact that the BMS-AS-D algorithm makes a multivariate decision on the whole set of proteins, while the FOHSIC uses a forward procedure which can lead to error accumulation. Indeed, if any detected biomarker in the sequence is false, then the final selected model is bound to be erroneous. Furthermore, the BMS-AS-D is also faster than the FOHSIC, as illustrated in Table 4. More precisely, Table 5 shows the error rate *τ* for the FOHSIC and the Bayesian algorithm for *P*=8 and different number of biomarkers. Conversely, the results proposed in Table 6 are obtained with the number of biomarkers fixed to *M*=4 while the number of proteins *P* is varied. As expected, the performance of the FOHSIC algorithm is degraded when increasing the number of proteins while the opposite is observed for the BMS-AS-D. Thus, even for large *P*, the Bayesian algorithm outperforms the FOHSIC.

#### Results for the noisy model

The performance of the BMS-AS-N and the BMS-AS-D algorithms is first studied as a function of the number of proteins *P* and the number of individuals *N*, for a fixed noise level. Then, the BMS-AS-N and the BMS-AS-D methods are compared for different noise conditions.

Table 7 reports the error rate *τ* (%) for the BMS-AS-N and the BMS-AS-D methods. The value of the error rate for the BMS-AS-D method is increased as compared to the results given in Table 1. This is due to the fact that the BMS-AS-D relies on a noiseless model, i.e., it processes the noisy data as if they were noiseless protein concentrations. That is why this result is expected, especially given the specified severe noise conditions (*E*(***Γ***_*ε*_)=10^−2^ **I**_*P*_). As a consequence, the performance of the BMS-AS-N method is significantly better than the BMS-AS-D one. Also, the difference in performance increases with the number of proteins, since for large sets of proteins, the number of candidate models increases and the estimation becomes more difficult.

**Table 7:** Noisy data: *τ* (%) for different values of *P*, *N*=1000

*P*	1	2	3	4
BMS-AS-D	16.82	32.784	48.740	63.686
BMS-AS-N	1.38	2.682	4.158	5.698

The performance of the BMS-AS-N method is also evaluated for several numbers of individuals: *N*=100,500, and 1000. Table 8 shows the results where it can be observed that for *N*=100, the error rate is higher; however, it does not exceed 13%. For the BMS-AS-D method, the results are even poorer because of the impact of the noise on smaller sample sizes.

**Table 8:** Noisy data: *τ* (%) for different valued of *N*, *P*=3

*N*	100	500	1000
BMS-AS-D	86.850	71.615	48.740
BMS-AS-N	12.477	5.781	4.158

To assess the importance of taking into account the noise in the model, the respective performances of the BMS-AS-D and the BMS-AS-N methods are also compared for different noise levels. We recall that the former is designed from the noiseless data model, while the latter specifically addresses the noisy data model. The noise power is measured by the mean value of the noise variance *E*(***Γ***_*ε*_), which is varied in the simulations. Table 9 shows the error rate for both methods as a function of this parameter: The BMS-AS-N method always outperforms the BMS-AS-D one. In the absence of noise, the BMS-AS-N method becomes equivalent to the optimal noiseless method. These results confirm the relevance of the method, especially for high noise levels.

**Table 9:** *τ* (%) for *N*=1000, *P*=3

*E*(***Γ***_*ε*_)(×**I**_*P*_)	10^−2^	10^−1^	1	10	10^2^
BMS-AS-D	48.740	12.304	3.064	0.813	0.291
BMS-AS-N	4.158	1.395	0.567	0.303	0.220

As a conclusion, the results confirm the good performance of the proposed BMS-AS-N method which is also not too computationally intensive by means of the analytical approximation of the posterior probabilities.

### Evaluation using the real data

The primary goal of this paper was to present a novel methodology for biomarker identification that relaxes classical simplifying assumptions on the data model and then to evaluate it on simulated data. Nevertheless, we had at our disposal a batch of real data^2^ and we used it to carry out a preliminary study of the BMS-AS-N method. The data are composed of 206 samples: 105 with the status ${\mathcal {H}}$ (including 76 patients from blood donors and 29 with negative colonoscopy), 101 with malignant tumor^3^, i.e. with status ${\mathcal {P}}$. The latter are structured as follows: 24 patients in the ‘stage one’ of the cancer, 26 patients in the ‘stage two’, 23 patients in the ‘stage three’, 25 patients in the ‘stage four’, and three missing values. The protein concentrations are obtained using the Bayesian inversion method developed in [34] from measurements of SRM spectra according to the methodology described in [35]. For each sample, the concentrations of 21 proteins are measured (14-3-3 protein sigma; 78-kDa glucose-regulated protein; protein S100-A11; calmodulin; calreticulin; peptidyl-prolyl cis-trans isomerase A; defensin-5; defensin-6; heat shock cognate 71 kDa protein; fatty acid-binding protein, intestinal; fatty acid-binding protein, liver (LFABP); stress-70 protein, mitochondrial; protein disulfide-isomerase (PDI); protein disulfide-isomerase A6 (PDIA6); phosphoglycerate kinase 1; retinol-binding protein 4; peroxiredoxin 5, mitochondrial; protein S100-A14; triosephosphate isomerase; villin-1 (Villin); Vimentin). Only one of the proteins in the sample, named LFABP, was previously identified by SRM as a biomarker [36]. To calculate the hyperparameters (18)–(21), empirical orders of magnitudes for (***m***_×_,***Γ***_×_) (e.g., *μ*g/ml) are used as specified: $E(\boldsymbol {m}_{\times })=10^{2}\mathbf {1}_{P^{\ast }},\ V(\boldsymbol {m}_{\times })=10^{3}\,\mathbf {I}_{P^{\ast }},\ E(\boldsymbol {\Gamma }_{\times })=10^{3}\,\mathbf {I}_{P^{\ast }},\ V(\boldsymbol {\Gamma }_{\times })=10\,\mathbf {I}_{P^{\ast }}$.

For this data set, the posterior probability is computed for each of the 2^21^ possible partitions according to (17) for the BMS-AS-N methods. Table 10 presents the four most probable partitions, with their probabilities. By far, the most probable partition (probability 0.9986) is: LFAPB is discriminant and the remaining 20 proteins are non-discriminant. The second most probable partition is: the whole set of protein is non-discriminant (probability 0.001361). The third and the fourth ones select two discriminant proteins and the 19 other are non-discriminant: (LFABP, Villin) and (LFABP, PDIA6), with probability smaller than 10^−6^. As a conclusion, the proteins are all declared as non-discriminant, except the LFAPB. This study confirms that our method correctly identifies the valid biomarker.

**Table 10:** The four most probable partitions, for real data *P*=21

Declared biomarker	LFABP	No biomarker	LFABP and Villin	LFAPB and PDIA6
Probability	9.986×10^−1^	1.361×10^−3^	9.762×10^−7^	8.297×10^−7^

Despite the large number of models to compare (about two millions candidate models), the computation time is just 1 h. This short computation time is made possible by the analytical calculation of the posterior probability, avoiding the use of extensive numerical integration methods such as for instance MCMC algorithms [24].

## Synthesis and perspectives

Biomarker discovery is a challenging task of the utmost interest for the diagnosis and prognosis of diseases. This paper presents a statistical approach based on variable selection. It is developed in a Bayesian framework that relies on an optimal strategy, i.e., the minimization of an error risk. Given *P* candidate proteins, the proposed procedure compares the probability of the 2^*P*^ partitions (subset of discriminant versus subset of non-discriminant proteins). The most a posteriori probable partition is finally retained and thus defines the selected variables. The main difficulty is the required integration with respect to all the unknown model parameters. An important contribution is to provide a closed-form expression of the probabilities for noiseless observations and a sensible approximation for noisy observations. The proposed method proved to be well-suited for variable selection in a complex context. Its effectiveness is assessed by a theoretical characterization and numerical studies (on simulated and real data) which are in accordance with the theoretical optimality. Furthermore, the proposed method compares favorably with the *t* test, the LASSO, the Battacharrya distance, and the FOHSIC.

From a methodological standpoint, several perspectives can be considered. Regarding the concentrations, non-Gaussian distributions, e.g., Gamma or Wishart models, could be relevant. Regarding the status, a possible development could account for possible errors in the given status. In this case, an additional level should be appended to the hierarchical Bayesian model. It would include a prior probability for an erroneous status.

As for the applicative perspectives, we plan to further take advantage of the performance of the method in other clinical data sets or in other biomedical fields (e.g., genomics, transcriptomics…). In addition, we also intend to make use of the method in other domains, for instance, in astrophysics (identification of pertinent features in order to classify galaxies), or for complex structures and industrial processes (identification of indicators for detection and diagnosis of damages or faults, analysis of fatigue and aging prevention,…).

## Endnotes

^1^ The knowledge about orders of magnitudes of the concentration values is acquired from the real data set provided by bioMérieux (Technology Research Department), France.

^2^ SRM measurements provided by bioMérieux (Technology Research Department), France

^3^ colorectal cancer

## Appendix 1

### Reduction of the concentration distribution

This section explains how the exponential arguments of the Gaussian distributions in (4) can be reformulated based on the empirical means and covariances of the concentrations, yielding relation (5). We have:

$${\begin{aligned} \prod_{n\in\mathcal{I}_{\mathcal{P}}} \mathcal{N}\left(\boldsymbol{x}_{n}^{+}; \boldsymbol{m}_{\mathcal{P}},\boldsymbol{\Gamma}_{\mathcal{P}}\right) &=\prod\limits_{n\in\mathcal{I}_{\mathcal{P}}} (2\pi)^{-P^{+}/2}\vert \boldsymbol{\Gamma}_{\mathcal{P}}\vert^{1/2}\\ &\exp\left[ -\left(\boldsymbol{x}_{n}-\boldsymbol{m}_{\mathcal{P}}\right)^{\mathrm{t}}\boldsymbol{\Gamma}_{\mathcal{P}}\left(\boldsymbol{x}_{n}-\boldsymbol{m}_{\mathcal{P}}\right)/2 \right] \\ &=(2\pi)^{-P^{+}N_{\mathcal{P}}/2} \vert \boldsymbol{\Gamma}_{\mathcal{P}}\vert^{N_{\mathcal{P}}/2}\\ &\exp\left[ -\sum_{n\in \mathcal{I}_{\mathcal{P}}}\left(\boldsymbol{x}_{n}-\boldsymbol{m}_{\mathcal{P}}\right)^{\mathrm{t}}\boldsymbol{\Gamma}_{\mathcal{P}}\left(\boldsymbol{x}_{n}-\boldsymbol{m}_{\mathcal{P}}\right)/2 \right] \end{aligned}} $$

The idea is to re-arrange the sum in the exponential as a function of the empirical mean $\bar {\boldsymbol {x}}_{\mathcal {P}}^{+}$ and the empirical variance $\bar {\mathbf {R}}_{\mathcal {P}}^{+}$. To this end, for the sake of calculation simplicity, we can write: $\boldsymbol {x}_{n}-\boldsymbol {m}_{\mathcal {P}}=\left (\boldsymbol {x}_{n}-\bar {\boldsymbol {x}}_{\mathcal {P}}^{+}\right) -\left (\bar {\boldsymbol {x}}_{\mathcal {P}}^{+}-\boldsymbol {m}_{\mathcal {P}}\right)$ and develop the sum of product. Then, using the fact that ***u*** ^t^ ***M*** ***u***=Tr(***u*** ^t^ ***M*** ***u***)=Tr(***M*** ***u*** ***u*** ^t^), the product is written as function of empirical mean and variance

22 $$ {\begin{aligned} \prod_{n\in\mathcal{I}_{\mathcal{P}}} & \mathcal{N}\left(\boldsymbol{x}_{n}^{+}; {\boldsymbol{m}}_{\mathcal{P}},{\boldsymbol{\Gamma}}_{\mathcal{P}}\right)= (2\pi)^{-P^{+}N_{\mathcal{P}}/2} \vert {\boldsymbol{\Gamma}}_{\mathcal{P}}\vert^{N/2} \\ & \exp\left[-\frac{N_{\mathcal{P}}}{2}\text{Tr}\left(\boldsymbol{\Gamma}_{\mathcal{P}} \left[\bar{\mathbf{R}}_{\mathcal{P}}^{+} + \left(\bar{\boldsymbol{x}}_{\mathcal{P}}^{+} -{\boldsymbol{m}}_{\mathcal{P}}\right)\left(\bar{\boldsymbol{x}}_{\mathcal{P}}^{+} -{\boldsymbol{m}}_{\mathcal{P}}\right)^{\mathrm{t}} \right] \right)\right] \end{aligned}}  $$

which allows easier handling of the Normal-Wishart prior.

## Appendix 2

### Wishart, Normal-Wishart, and matrix variate t-distribution

#### Wishart

The Wishart density probability function for a *P*×*P* matrix ***Γ*** is driven by two parameters: a degree of freedom *ν* (real and larger than *P*−1) and a matrix ***Λ*** (positive and definite) referred to as the scale matrix. It reads

$$\begin{aligned} \mathcal{W}(\boldsymbol{\Gamma} \,;\, \boldsymbol{\Lambda},\nu) &= {\mathcal{KW}}^{-1}~ \text{det}\left[{\boldsymbol{\Gamma}}\right]^{(\nu-P-1)/2}\\ &\quad\text{exp}\left[ - \text{Tr}\left[\boldsymbol{\Gamma} \boldsymbol{\Lambda}^{-1}\right] / 2 \right] \end{aligned} $$

The normalizing constant ${\mathcal {KW}}$ depends on *ν* and ***Λ***:

$${\mathcal{KW}} = {\mathcal{KW}}(\nu,\boldsymbol{\Lambda}) =2^{\nu P /2} \, \text{det}\left[\boldsymbol{\Lambda}\right]^{\nu /2} \, \gamma_{P}(\nu/2) $$

where *γ*_*P*_ is the *P*-dimensional Gamma function.

#### Normal-Wishart

For a couple (***m***,***Γ***), where ***m*** is a *P*-dimensional vector and ***Γ*** a *P*×*P* matrix, the Normal-Wishart density is controled by four parameters *ν*,*η*,***μ***,***Λ***. It reads:

23 $$ { \begin{aligned} \mathcal{N}\mathcal{W}(\boldsymbol{m},\boldsymbol{\Gamma} \,;\, \nu,\eta, \boldsymbol{\mu}, \boldsymbol{\Lambda}) &=\mathcal{KNW}^{-1}~ \text{det}\left[\boldsymbol{\Gamma}\right]^{(\nu-P)/2} \\ & \quad\text{exp}\left[-\left[\text{Tr}\left[{\boldsymbol{\Gamma} \boldsymbol{\Lambda}^{-1}}\right]\right.\right.\\ &\left.\left.\quad+ \eta (\boldsymbol{m} - \boldsymbol{\mu})^{\mathrm{t}}\boldsymbol{\Gamma}(\boldsymbol{m} - \boldsymbol{\mu})\right]/2\right] \end{aligned}}  $$

and the normalizing constant is:

24 $$  \begin{aligned} \mathcal{KNW} &= \mathcal{KNW}(\nu,\eta,\boldsymbol{\mu},\boldsymbol{\Lambda}) = (2\pi)^{P/2} \, 2^{\nu P /2} \, \eta^{-P/2}\\ &\quad\text{det}\left[{\boldsymbol{\Lambda}}\right]^{\nu /2} \, \gamma_{P}(\nu/2) \,, \end{aligned}  $$

and it does not depend on ***μ*** (that is a position parameter).

#### Matrix variate t-distribution

The random matrix ***T*** of dimension (*P*×*N*) is said to have a matrix variate t-distribution with parameters ***M***,***Σ***,***Ω***, and *q* if its probability density function is given by

$$\begin{array}{@{}rcl@{}} \mathcal{T}_{P,N}(q,\boldsymbol{M},q\boldsymbol{\Sigma},\boldsymbol{\Omega}) &=& \frac{\gamma_{P}\left(\left[q+N+P-1\right]/{2}\right)}{\gamma_{P}\left(\left[{P+p-1}\right]/{2}\right)} ~ \pi^{-NP/2} \\ &&\vert \boldsymbol{\Sigma}{\vert}^{-N/2}~ \vert\boldsymbol{\Omega}{\vert}^{-P/2} ~ \vert \boldsymbol{I}_{P}+\boldsymbol{\Sigma}^{-1}(\boldsymbol{T}-\boldsymbol{M})\\&&\boldsymbol{\Omega}^{-1} (\boldsymbol{T}-\boldsymbol{M})^{\mathrm{t}}{\vert}^{-\left[{q+N+P-1}\right]/{2}} \end{array} $$

where ***T*** and ***M*** are *P*×*N* matrices, ***Ω*** and ***Σ*** are positive-definite matrices with respective sizes *N*×*N* and *P*×*P* and *q*>0.

When *q* tends to infinity, the distribution of ***T*** tends to a Gaussian distribution with mean ***M*** and covariance ***Σ***⊗***Ω*** that is to say $\mathcal N(\boldsymbol {T}\, ; \,\boldsymbol {M},\boldsymbol {\Sigma }\otimes \boldsymbol {\Omega })$, where ⊗ is the Kronecker product.
